# Genetic Potential of *Dissulfurimicrobium hydrothermale*, an Obligate Sulfur-Disproportionating Thermophilic Microorganism

**DOI:** 10.3390/microorganisms10010060

**Published:** 2021-12-28

**Authors:** Stéven Yvenou, Maxime Allioux, Alexander Slobodkin, Galina Slobodkina, Mohamed Jebbar, Karine Alain

**Affiliations:** 1 Laboratoire de Microbiologie des Environnements Extrêmes LM2E, Université de Brest, CNRS, IFREMER, IRP 1211 MicrobSea, IUEM, Rue Dumont d’Urville, F-29280 Plouzane, France; steven.yvenou@univ-brest.fr (S.Y.); maxime.allioux@univ-brest.fr (M.A.); Mohamed.Jebbar@univ-brest.fr (M.J.); 2 Research Center of Biotechnology of the Russian Academy of Sciences, Winogradsky Institute of Microbiology, 117312 Moscow, Russia; aslobodkin@hotmail.com (A.S.); gslobodkina@mail.ru (G.S.)

**Keywords:** sulfur disproportionation, hydrothermal vent, genomics, thermophile

## Abstract

The biochemical pathways of anaerobic sulfur disproportionation are only partially deciphered, and the mechanisms involved in the first step of S^0^-disproportionation remain unknown. Here, we present the results of sequencing and analysis of the complete genome of *Dissulfurimicrobium hydrothermale* strain Sh68^T^, one of two strains isolated to date known to grow exclusively by anaerobic disproportionation of inorganic sulfur compounds. *Dissulfurimicrobium hydrothermale* Sh68^T^ is a motile, thermophilic, anaerobic, chemolithoautotrophic microorganism isolated from a hydrothermal pond at Uzon caldera, Kamchatka, Russia. It is able to produce energy and grow by disproportionation of elemental sulfur, sulfite and thiosulfate. Its genome consists of a circular chromosome of 2,025,450 base pairs, has a G + C content of 49.66% and a completion of 97.6%. Genomic data suggest that CO_2_ assimilation is carried out by the Wood–Ljungdhal pathway and that central anabolism involves the gluconeogenesis pathway. The genome of strain Sh68^T^ encodes the complete gene set of the dissimilatory sulfate reduction pathway, some of which are likely to be involved in sulfur disproportionation. A short sequence protein of unknown function present in the genome of strain Sh68^T^ is conserved in the genomes of a large panel of other S^0^-disproportionating bacteria and was absent from the genomes of microorganisms incapable of elemental sulfur disproportionation. We propose that this protein may be involved in the first step of elemental sulfur disproportionation, as S^0^ is poorly soluble and unable to cross the cytoplasmic membrane in this form.

## 1. Introduction

Chemolithotrophic microorganisms derive the energy necessary for their growth by transforming mineral species by oxidation–reduction reactions. They transform a wide variety of mineral species in both oxidized and reduced states, which can be used as electron donors or as terminal electron acceptors, respectively. In contrast, chemoorganotrophic microorganisms oxidize or ferment organic compounds to gain energy for growth. With the exception of fermentation, all these metabolisms are based on the use of a reduced compound (organic or inorganic) as an electron donor and a more oxidized one as a terminal electron acceptor.

The disproportionation (=dismutation) of inorganic sulfur compounds (ISC) is to some extent outside this general framework. It is a scarcely studied metabolism, found in three bacterial phyla (*Firmicutes*, *Nitrospirota* and *Desulfobacterota* (including *Thermodesulfobacteriales* and various orders previously classified in the *Deltaproteobacteria*)), which is distinguished by the ability to use only a single mineral species (S^0^, S_2_O_3_^2−^, SO_3_^2−^) as electron donor and acceptor [1,2]. Discovered in 1987 [3], it is also called inorganic fermentation [4]. The disproportionation of elemental sulfur (Equation (1)), thiosulfate (Equation (2)) and sulfite (Equation (3)) are, respectively, described by the following equations:

4S^0^ + 4H_2_O = SO_4_^2−^ + 3HS^−^ + 5H^+^ (1)

S_2_O_3_^2−^ + H_2_O = SO_4_^2−^ + HS^−^ + H^+^ (2)

4SO_3_^2−^ + H^+^ = 3SO_4_^2−^ + HS^−^ (3)

This metabolism, which has already been found in bacteria from various anoxic environments (marine sediments, hydrothermal vents, soda lakes, freshwater basins and others), seems to be widespread around the globe [5]. However, the extent, the importance and the contribution of this metabolism to the sulfur cycle are not yet known, for three main reasons: (i) first, there are no molecular markers specific for this metabolism, which does not allow the use of bioinformatics-based methods. (ii) Second, there are a limited number of sulfur-disproportionating bacteria that have been identified so far, and this ability has been experimentally demonstrated only for 42 strains [5]. (iii) Third, the products of ISC disproportionation are sulfate and sulfide, which makes it difficult to distinguish between sulfur disproportionation from sulfide or sulfur oxidation and from sulfate reduction. This means that ISC disproportionation is not recognized as such and is not sufficiently considered and integrated into current biogeochemical models.

Research conducted to date suggests that there are different pathways for sulfur disproportionation [1]. No complete pathway for elemental sulfur disproportionation has been described, although several studies have been performed to elucidate this [6,7,8,9,10]. In particular, it is not known which enzymes/mechanisms participate in the first step of S^0^-disproportionation. In the current state of knowledge, most of the enzymes identified as being involved in the disproportionation process are those in the reducing branch of the reaction and are shared with those of the dissimilatory sulfate reduction pathway. For this reason, no specific molecular markers for this metabolism have been identified so far. Most sulfur-disproportionating bacteria are capable of producing energy by another metabolic reaction (i.e., dissimilatory sulfate reduction, sulfite reduction, thiosulfate reduction, sulfur reduction, dissimilatory nitrate reduction to ammonium) and disproportionation could be used as an alternative or accessory metabolism (for maintenance energy production) by some taxa [1]. Up to date, only two strains are known to use the ISC-disproportionation as the only catabolic pathway [11,12]. One of them is *Dissulfurimicrobium hydrothermale* strain Sh68^T^. This strain is a thermophilic, anaerobic chemolithoautotroph belonging to the phylum *Desulfobacterota* (previously classified as a *Deltaproteobacteria*) [13], which was isolated in 2016 from a hydrothermal basin at Uzon Caldera, Kamchatka, in Russia [12]. The physiological characterization showed that this strain was only able to grow by ISC-disproportionation (S^0^, S_2_O_3_^2−^, SO_3_^2−^) and did not reduce sulfate, nitrate, Fe(III) citrate, ferrihydrite, AQDS, fumarate or oxygen with acetate, lactate, ethanol, pyruvate, fumarate, maleinate, malate, succinate, peptone or H_2_ as electron donors, under the tested conditions [12]. This strain is phylogenetically distant from other sulfur-disproportionating taxa. Its closest phylogenetic relative is *Dissulfuribacter thermophilus* strain S69^T^, from the same class, sharing only 90% sequence similarity of the 16S rRNA gene.

The objective of this study was to analyze the genome of *Dissulfurimicrobium hydrothermale* strain Sh68^T^ and the metabolic pathways encoded by this organism, in particular those of the sulfur cycle. Comparative genomic analyses between sulfur-disproportionators and non-sulfur-disproportionators were performed to seek specific markers for this catabolic pathway.

## 2. Materials and Methods

### 2.1. Culture, DNA Extraction and Genome Assembly

*Dissulfurimicrobium hydrothermale* strain Sh68^T^ was grown at 50 °C, in a culture medium (pH 6, NaCl 1.5% (*w*/*v*)) dedicated to the cultivation of elemental sulfur disproportionators supplemented with 90 mM of Fe(OH)_3_ as sulfide scavenger, under a CO_2_ 100% atmosphere (1 bar), as described elsewhere [14]. Genomic DNA was extracted with a standard PCI (Phenol: Chloroform: Isoamyl Alcohol (25:24:1) protocol [15], after dissolving Fe(OH)_3_ with a solution composed of sodium dithionite (50 g.L^−1^), acetic acid (0.35 M) and sodium citrate (0.2 M) [16]. The complete genome sequencing of strain Sh68^T^ was determined by hybrid sequencing, combining short and long read sequencing. Short read DNA sequencing was conducted by Fasteris SA (Plan-les Ouates, Switzerland) using the Illumina nanoMiSeq technology (2 × 150 bp paired-reads, Nano V2 chemistry). In parallel, the Oxford nanopore MinION technology (R9 Flow Cell, Rapid Sequencing kit) was applied to obtain long reads. Quality control of the short and long reads were then performed using FastQC (v0.11.9; https://www.bioinformatics.babraham.ac.uk/projects/fastqc/, accessed on 7 December 2021 [17] and filtered using fastp (v0.22.0; https://github.com/OpenGene/fastp, accessed on 7 December 2021) [18].

High quality reads were then assembled by using Unicycler for de novo hybrid assembly (v0.4.9; https://github.com/rrwick/Unicycler), and its dependencies (spades.py v3.13.0; makeblastdb v2.6.0+; tblastn v2.6.0+; bowtie2-build v2.4.4; bowtie2 v2.4.4; samtools v1.11; java v11.0.9.1; pilon v1.23 accessed on 7 December 2021) [19], N50 and other genome assembly statistics were obtained with Quast (Galaxy Version 5.0.2 + galaxy2) [20]. Genome completeness and potential contamination were controlled with CheckM (v1.1.3; https://ecogenomics.github.io/CheckM/ accessed on 7 December 2021) [21]. The average coverage of the whole genome was calculated with the following formula: coverage = (number of reads × read length)/total genome size.

### 2.2. Genome Annotation

Genome was analyzed and annotated with the following software/pipelines, with default parameters and associated databases: the fast annotation software Prokka (v1.13; https://github.com/tseemann/prokka accessed on 7 December 2021) [22], the MicroScope Microbial Genome Annotation and Analysis Platform (MaGe) (https://mage.genoscope.cns.fr/microscope/home/index.php accessed on 7 December 2021) [23] and the Prokaryotic Genome Annotation Pipeline from NCBI (PGAP) (https://www.ncbi.nlm.nih.gov/genome/annotation_prok/ accessed on 7 December 2021) [24]. Functional annotations of predicted CDSs were compared on UniProtKB against the UniProtKB reference proteomes and the Swiss-Prot database (https://www.uniprot.org/ accessed on 7 December 2021). Classification of genes into COG (Cluster of Orthologous Groups) and eggNOG groups were performed with MaGe. Identification and classification of CRISPR-Cas systems were performed by using the CRISPRCasFinder webserver (https://crisprcas.i2bc.paris-saclay.fr/ accessed on 7 December 2021) [25]. The heatmap was constructed on Anvi’o (v7.1) from metabolic predictions made by KEGG (KOfam) (https://merenlab.org/software/anvio/ accessed on 7 December 2021) [26]. The circular map of the genome was generated with the ‘GCView’ tool of MaGe. Specific markers of S^0^ disproportionation were sought by comparing the results of PGAP annotations within a set of species genome for which sulfur disproportionation ability was tested and whose genomes were available online on NCBI and annotated by PGAP. Results were further confirmed by comparing protein sequence homology with blastp (v.2.12.0), (https://blast.ncbi.nlm.nih.gov/Blast.cgi?PAGE=Proteins accessed on 7 December 2021), using the set of microbial genomes selected for this analysis as a reference database. This set comprised the genomes of *Dissulfuribacter thermophilus* S69^T^ (ASM168733v1) [27], *Thermosulfurimonas marina* SU872^T^ (ASM1231758v1) [28], *Thermosulfurimonas dismutans* S95^T^ (ASM165258v1) [14], *Dissulfurirhabdus thermomarina* SH388^T^ (ASM1297923v1) [29], *Caldimicrobium thiodismutans* TF1^T^ (ASM154827v1) [11], *Desulfobulbus propionicus* DSM 2032^T^ (ASM18688v1) [30], *Desulfofustis glycolicus* DSM 9705^T^ (IMG-taxon 2585428080 annotated assembly) [31], *Desulfonatronospira thiodismutans* ASO3-1^T^ (ASM17443v1) [32], *Desulfonatronum lacustre* DSM 10312^T^ (ASM51926v1) [33], *Desulfonatronum thioautotrophicum* ASO4-1^T^ (ASM93474v1) [34], *Desulfonatronum thiodismutans* MLF1^T^ (ASM71747v2) [35], *Desulfonatronum thiosulfatophilum* ASO4-2^T^ (IMG-taxon 2596583601 annotated assembly) [34], *Desulfolutivibrio sulfodismutans* (previously referred as *Desulfovibrio sulfodismutans*) DSM 3696^T^ (ASM1337645v1) [36], *Desulfocapsa sulfoexigens* DSM 10523^T^ (ASM34139v1) [37], *Desulfurella amilsii* TR1^T^ (ASM211942v1) [38], *Desulfurivibrio alkaliphilus* AHT 2^T^ (ASM9220v1) [39], *Dethiobacter alkaliphilus* AHT 1^T^ (ASM17441v1) [39], *Thermosulfuriphilus ammonigenes* ST65^T^ (ASM1120745v1) [40], *Thermodesulfatator atlanticus* DSM 21156^T^ (ASM42158v1) [41], *Desulforhopalus vacuolatus* DSM 9700^T^ (ASM1691850v1) [42], *Dissulfurispira thermophila* T55J^T^ (ASM1470123v1) [43], and *Thermodesulfatator indicus* DSM 15286^T^ (ASM21779v1) [44].

### 2.3. Data Availability

The complete genome sequence of *Dissulfurimicrobium hydrothermale* Sh68^T^ was deposited in GenBank/DDBJ/EMBL databases under the accession numbers CP085041 for the genome, and PRJNA769390 for the BioProject.

## 3. Results and Discussion

### 3.1. General Characteristics of Genome

**Figure 1:**
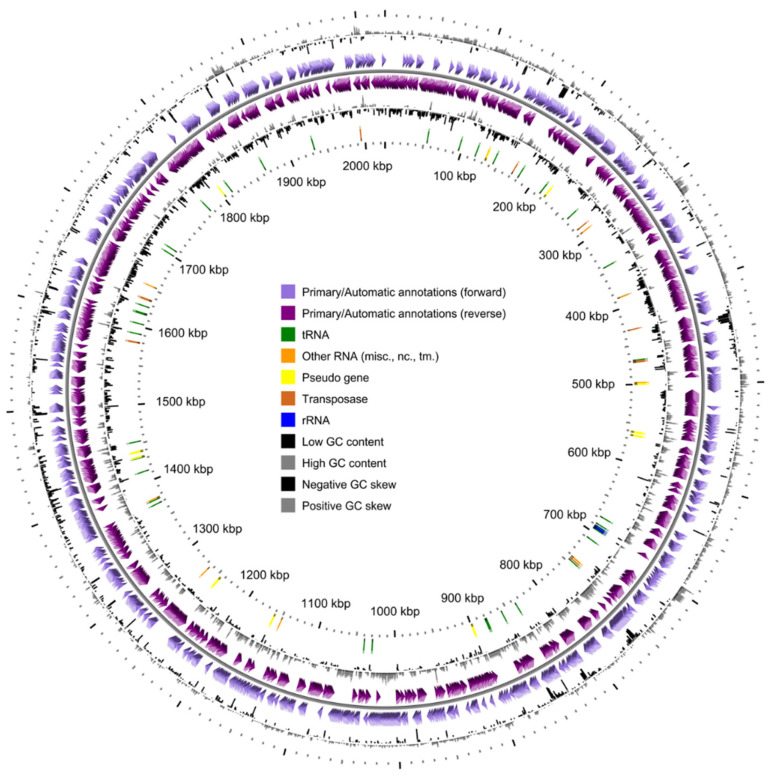
Circular map of the chromosome in the genome of *Dissulfurimicrobium hydrothermale* Sh68^T^. From the outside to the center: CDSs position on the forward and reverse strands, DNA GC skew, DNA G+C content, tRNAs—rRNAs—other RNAs-pseudogenes and transposases.

MinION sequencing provided 163,040,184 bases and Illumina MiSeq 102,590,792 nucleotides. The complete genome of *Dissulfurimicrobium hydrothermale* strain Sh68^T^ obtained by hybrid assembly, consists of one unique contig of 2,025,450 nucleotides and has a GC content of 49.66% (Table 1, Figure 1). No plasmids were detected. Annotation with MaGe predicted a protein coding density of approximately 91.09%. Annotation with PGAP resulted in prediction of 1875 CDSs, 1824 of which were protein-coding genes, 47 tRNA genes for all standard amino acids, 54 RNA genes and 51 pseudogenes (Table 1). Genome contains one *rrn* operon of 5S-16S-23S rRNA genes. Considering only short read sequences, the genome coverage is about 50.6×, and reaches 131.15× with both long and short reads (723,264 used reads in total).

CheckM analysis estimated the genome to be 97.597% complete (6 marker(s) were missing) and hypothetical contamination to be 1.19048% (2 marker(s) were duplicated) (Table 1). One CRISPR-Cas type Ic gene cluster and two other CRISPR loci were predicted with CRISPRCasFinder.

In total, 84.36% of the CDSs could be assigned to at least one COG group, and 89.30% of the CDSs were classified into at least one eggNOG group. The main COG categories and the main eggNOG groups (encompassing more than 2% of the CDSs) were respectively related to cell wall/membrane/envelope biogenesis (M)(7.7% for COG and 7.3% for eggNOG), translation, ribosomal structure and biogenesis (J)(7.4% and 7.1% for COG and eggNOG, respectively), signal transduction mechanisms (T)(6.8% and 6.0%), cell motility (N)(4.8% and 2.4%), intracellular trafficking, secretion, and vesicular transport (U)(4.5% and 2.9%), posttranslational modification, protein turnover, chaperones (O)(4.3% and 4.2%).

### 3.2. Carbon Metabolism

Physiological studies demonstrated that *Dissulfurimicrobium hydrothermale* Sh68^T^ can grow autotrophically utilizing CO_2_/HCO_3_^−^ as a sole carbon source [12]. Different annotation software used and the functional annotations with the UniProtKB database predicted the presence of a complete Wood-Ljungdhal pathway (=reductive acetyl-CoA pathway) for autotrophic carbon fixation. This non-cyclic pathway, also present in several sulfur-disproportionating strains [5,9,45], and sulfate reducing bacteria [46,47], enables the production of acetyl-CoA through the reduction of two CO_2_ molecules [46]. This pathway includes a formate dehydrogenase (LGS26_01375; LGS26_01895; LGS26_01380), a formate-tetrahydrofolate ligase (LGS26_01960), a bifunctional methylenetetrahydrofolate dehydrogenase/cyclohydrolase (LGS26_01955), a 5-methyltetrahydrofolate corrinoid/iron-sulfur protein methyltransferase (LGS26_01905), a carbon-monoxide dehydrogenase (LGS26_01950), a 5,10-methylenetetrahydrofolate reductase (LGS26_08700; LGS26_08025) and a CO-methylating acetyl-CoA synthase (LGS26_01915).

**Figure 2:**
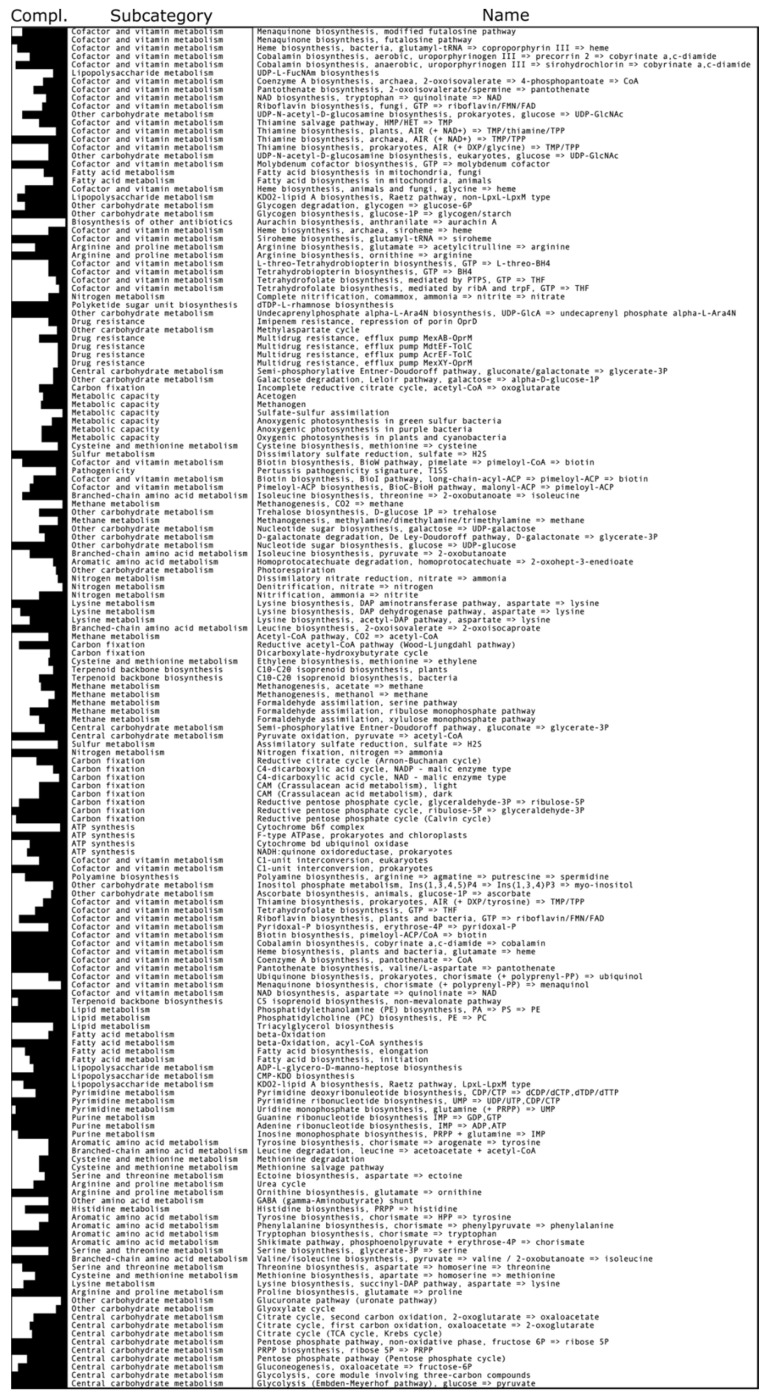
Summary graphical representation of predicted metabolic pathways according to the KEGG database and their completeness, generated by Anvi′o. The black bars on the left indicate the degree of completion of metabolic pathways according to the KEGG classification: the longer the bars are, the more complete the metabolic pathways. The gluconeogenesis and Wood–Ljungdahl pathways, which are incomplete according to KEGG, were predicted as complete by the MicroCyc tool on MaGe and by PGAP before functional confirmation against UniProtKB.

Acetyl-CoA molecules formed by this carbon fixation pathway can then be used as precursor metabolites for lipid synthesis or converted to pyruvate before being converted to carbohydrate by gluconeogenesis. This genome also codes for a pyruvate synthase (LGS26_04520; LGS26_04525) that theoretically allows the transformation of acetyl-CoA into pyruvate (Figure 2). In addition, we have also identified a complete glycolysis pathway in the genome (Figure 2). However, because physiological studies conducted on *Dissulfurimicrobium hydrothermale* Sh68^T^ showed that this strain could not grow by glucose fermentation [12], it is likely that this pathway is used in the direction of gluconeogenesis. The gluconeogenesis path includes a phosphopyruvate hydratase (also called enolase) (LGS26_04420), a phosphoglycerate mutase (LGS26_04575; LGS26_02480), an ATP-dependent 6-phosphofructokinase (LGS26_09610), a fructose bisphosphate aldolase (LGS26_02340; LGS26_03835), a fructose-1,6-bisphosphatase class 1 (LGS26_09055), a fructose-1,6-bisphosphatase (LGS26_02360), a glyceraldehyde-3-phosphate dehydrogenase (LGS26_02375; LGS26_08515; LGS26_08525), a pyruvate kinase (LGS26_05830; LGS26_02335), a phosphoenolpyruvate synthase (LGS26_08740; LGS26_08765; LGS26_08745; LGS26_06715; LGS26_08520), a glucose-6-phosphate isomerase (LGS26_05870) and a phosphoglycerate kinase (LGS26_08510; LGS26_02380).

Prokka also predicted the presence of a pyruvate carboxylase (LGS26_05425), which is involved in the conversion of pyruvate into oxaloacetate.

Based on the predictions obtained from MaGe, PGAP and Prokka, the Calvin–Ben–on-Bassham and the reverse tricarboxylic acid (rTCA) cycles for carbon fixation appear incomplete (Figure 2).

The genome also encodes a complete formaldehyde oxidation V pathway (tetrahydrofolate pathway, which is completely reversible) including a formate-tetrahydrofolate ligase (LGS26_01960) and a methenyltetrahydrofolate cyclohydrolase/methylenetetrahydrofolate dehydrogenase (LGS26_01955). These enzymes are involved in the conversion of formate into 5,10-methylenetetrahydrofolate. An acetyl-coenzyme A synthetase (= acetate-CoA ligase) (LGS26_05510) is also encoded in the genome allowing the conversion of acetate into acetyl-CoA. The genome encodes also a formate dehydrogenase (LGS26_01380), which might allow the oxidation of formate into CO_2_. Alternatively, this pathway could work in the opposite direction, in the direction of converting formate into 5,10-methylenetetrahydrofolate, which could enter the serine cycle where its carbon could be used for biosynthesis.

### 3.3. Nitrogen Metabolism

The genome of *Dissulfurimicrobium hydrothermale* encodes a full set of genes for nitrogen fixation [48,49], including a nitrogenase molybdenum-iron protein beta chain (nifK) (LGS26_09450), a nitrogenase molybdenum-iron protein alpha chain (nifD) (LGS26_09455), a nitrogenase iron protein (nifH) (LGS26_09470), as well as a nitrogenase iron-molybdenum cofactor biosynthesis protein (LGS26_09445), a nitrogenase molybdenum-iron protein (nifN) (not reviewed on UniProtKB) (LGS26_09440), a nitrogen regulatory protein P-II (LGS26_09460 (glnB); LGS26_09465; LGS26_02655), and a nif-specific regulatory protein (nifA) (LGS26_02035; LGS26_09475). These results suggest that strain Sh68^T^ may have the ability to fix nitrogen into ammonia. The nitrogenase complex is also encoded in the genomes of other thermophilic sulfur-disproportionating bacteria of hydrothermal origin, namely *Thermosulfurimonas marina* and *Thermosulfuriphilus ammonigenes*.

We did not find any strong evidence for a catabolism based on the reduction of nitrogen compounds (denitrification or dissimilatory nitrate reduction to ammonium) in the genome of *D. hydrothermale*. This is consistent with the physiological studies, which showed that this strain is not able to reduce nitrate with a wide range of electron donors (acetate, lactate, ethanol, pyruvate, fumarate, maleinate, malate, succinate, peptone or H_2_) [12]. However, we have identified other genes involved in nitrogen metabolism, including ammonia/ammonium transporters (LGS26_02650), a hydroxylamine reductase (LGS26_05720) and a hydroxylamine oxidase (LGS26_01085). The genome codes also for a glutamine synthetase (glutamate-ammonia ligase) (LGS26_02665) allowing theoretically the conversion of glutamate into glutamine by fixation of NH_3_ and use of ATP. A glutamine amidotransferase is also present (LGS26_03405). Based on these genomic predictions, *D. hydrothermale* may have the genetic potential to fix nitrogen and to import ammonia, which are essential for the biosynthesis of macromolecules such as amino acids.

### 3.4. Hydrogen Metabolism

Physiological investigations have shown that this strain is unable to grow by hydrogen oxidation (with sulfate, nitrate, Fe (III) citrate, ferrihydrite, AQDS, fumarate or oxygen as terminal electron acceptors). However, its genome encodes several hydrogenase-related proteins, including maturation factors, formation chaperones and hydrogenase subunits. It encodes notably a catalytic subunit of a [NiFe] Group 1c (LGS26_08980) according to HydDB software. In addition, this genome codes also for several non-catalytic hydrogenase subunits, as a hydrogenase small subunit (LGS26_08975), a Ni/Fe-hydrogenase b-type cytochrome subunit (LGS26_08985), a HypC/HybG/HupF family hydrogenase formation chaperone (LGS26_08995), a hydrogenase formation protein HypD (LGS26_09000), a hydrogenase expression/formation protein HypE (LGS26_09005), a HyaD/HybD family hydrogenase maturation endopeptidase (LGS26_09010), a hydrogenase maturation nickel metallochaperone HypA (LGS26_09015), a hydrogenase nickel incorporation protein HypB (LGS26_09020), and three hydrogenase iron-sulfur subunits (LGS26_08855; LGS26_01930; LGS26_01940). It is not known what role these proteins play in this strain, which seems unable to oxidize hydrogen.

### 3.5. Sulfur Metabolism

**Figure 3:**
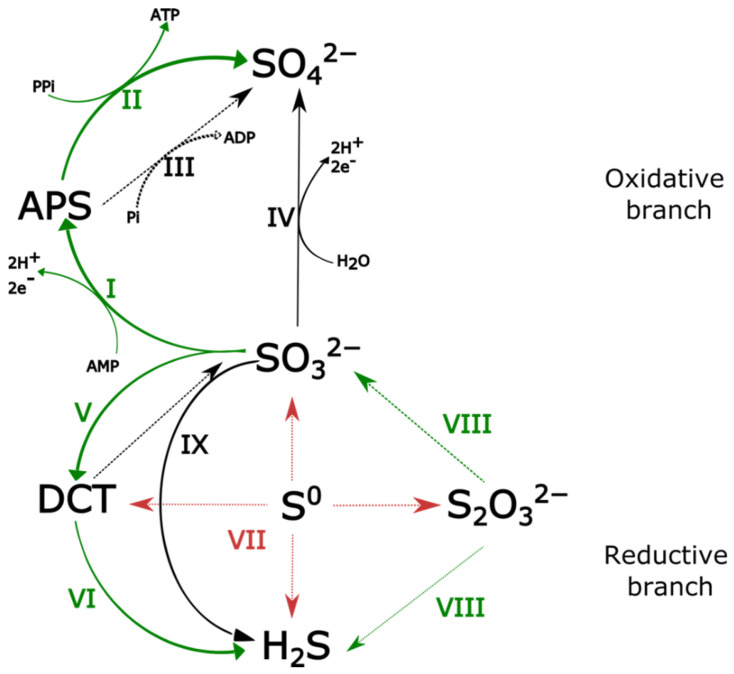
Schematic representation of the hypothetical involvement of enzymes encoded in the *D. hydrothermale* genome in a ISC-disproportionation pathway, based on functional analyses and hypotheses made elsewhere [1,4,6,51,52]. Bold green lines indicate that the enzyme is encoded in the genome. Dotted thin green lines indicate that the enzyme is putatively encoded in the genome. Thin black lines indicate that the enzyme was not predicted in the genome. Dotted thin red arrows indicate hypothetical reactions for which no enzyme has been identified so far. Legend: APS, Adenosine PhosphoSulfate; DCT, DsrC-Trisulfide. Enzymes encoded in *D. hydrothermale* genome: I, APS reductase (AprAB); II, ATP sulfurylase (=sulfate adenylyltransferase; Sat); V, DsrAB/DsrC complex; VI, DsrMKJOP system. Enzymes putatively encoded in *D. hydrothermale* genome: VIII, Thiosulfate reductase. Unresolved reactions: VII, unknown proteins. Enzymes not predicted in *D. hydrothermale* genome: III, Adenylylsulfate:phosphate adenylyltransferase (=ADP sulfurylase; Apt); IV, Sulfite oxidoreductase (SOR); IX, Sulfite reductase.

Physiological experiments demonstrated that *Dissulfurimicrobium hydrothermale* is able to grow by disproportionation of elemental sulfur, thiosulfate and sulfite [12] but not by sulfate reduction. However, a full pathway for dissimilatory sulfate reduction [50] is encoded in the genome of *D. hydrothermale*. Indeed, the genome encodes a sulfate adenylyltransferase (=ATP sulfurylase, Sat) (LGS26_04225), an adenylyl-sulfate reductase subunit beta (AprB) (LGS26_04230), an adenylyl-sulfate reductase subunit alpha (AprA) (LGS26_04235), a manganese-dependent inorganic pyrophosphatase (LGS26_02945), the four subunits of the dissimilatory-type sulfite reductase, namely DsrA, DsrB, DsrC and DsrD (LGS26_00980; LGS26_00985; LGS26_05895; LGS26_00990), a complete sulfate reduction electron transfer complex DsrMKJOP (LGS26_01035; LGS26_01030; LGS26_01025; LGS26_01020; LGS26_01015) and a complete quinone-interacting membrane-bound oxidoreductase complex (APS reductase-associated electron transfer complex) QmoABC (LGS26_04240; LGS26_04245; LGS26_04250). The last two operons, DsrMKJOP and QmoABC, were identified on the basis of protein sequence homology with *Dissulfuribacter thermophilus* proteins. This is not the first example of a sulfur-disproportionating strain that has a complete sulfate reduction pathway but seems unable to respire sulfate [1,8]. Some of these enzymes (Sat, AprAB, DsrABD, DsrC, DsrMK) could be involved in the reducing branch of the disproportionation pathway of inorganic sulfur compounds, as has been shown in other bacterial taxa [1,4] (Figure 3).

A recent study showed that a group of genes (YTD cluster) is consistently present in non-sulfate-reducing microorganisms capable of ISC-disproportionation and suggested that this cluster could be a genetic marker for ISC-disproportionation [2]. This YTD gene cluster is typically composed of the following genes: an *yedE*-related gene, a *dsrE*-related gene, a *tusA* gene, and two genes coding for conserved hypothetical proteins (CHPs) [2]. This YTD gene cluster is encoded in the genome of *Dissulfurimocrobium hydrothermale* Sh68^T^, which is a non-sulfate reducing organism capable of growth by ISC-disproportionation. Indeed (i) a sulfurtransferase TusA (LGS26_02870) protein is encoded and shares the highest protein sequence homology with TusA from *Dissulfurirhabdus thermomarina*, (ii) a YeeE/YedE family protein (LGS26_02865) is present and shares the highest protein sequence homology with its homolog in *Dissulfurirhabdus thermomarina*, (iii) a DsrE family protein (LGS26_02875) is encoded, sharing highest protein sequence homology with *Desulfosarcina widdelii* and *Desulfobacterium vacuolatum* proteins. These three CDS are followed by two uncharacterized proteins (LGS26_02880; LGS26_02885). Another gene coding for a sulfurtransferase TusA family protein (LGS26_05795) and genes coding for DsrE family proteins (LGS26_08090; LGS26_05760; LGS26_07510) are also encoded in the genome, but their function is still unknown, although they may be involved in sulfur metabolism.

Furthermore, a heterodisulfide reductase subunit A (LGS26_08860) and several molybdopterin-dependent oxidoreductases are encoded in the genome of *D. hydrothermale*. In brief, these molybdopterin-dependent oxidoreductases include: an oxidoreductase (LGS26_05355), which is most likely a tetrathionate reductase subunit A according to UniProtKB; an oxidoreductase (LGS26_06635), which is most likely a polysulfide/thiosulfate, catalytic subunit A according to UniProtKB; a protein (LGS26_09210) annotated as a thiosulfate reductase by Prokka and UniProtKB; and a molybdopterin-containing PhsA subunit of the thiosulfate reductase (LGS26_09210). Molybdopterin oxidoreductases are present in most genomes of ISC-disproportionators [1,9]. Thiosulfate reductase could be involved in the cleavage of thiosulfate into sulfite and hydrogen sulfide [4] (Figure 3). A rhodanese-like sulfurtransferase (LGS26_05360) was also predicted in the genome of *D. hydrothermale*, sharing protein sequence homology with *Hyella patelloides.* Rhodanese-type sulfurtransferases were found to be predominant in *Desulfurella amilsii* proteomes obtained under sulfur disproportionation conditions [10].

Finally, a sulfurtransferase-like selenium metabolism protein YedF (LGS26_04730), generally associated with the sulfur oxidation pathway, was predicted, but no other sulfur oxidation pathway enzymes such as sulfur oxygenase reductase (Sor) or Sox-associated proteins were found in this genome.

In summary, the analysis of sulfur metabolism revealed the presence of a complete set of genes of the dissimilatory sulfate reduction pathway, of the YTD gene cluster, of molybdopterin oxidoreductases and of a rhodanese-like sulfurtransferase, which could all or in part be involved in the disproportionation of inorganic sulfur compounds (Figure 3).

### 3.6. Motility and Pili

Cultivation experiments combined with transmission electron microcopy showed that *Dissulfurimicrobium hydrothermale* Sh68^T^ is a motile bacterium bearing a polar flagellum [12]. Thirty-one proteins related to flagella have been predicted by PGAP in the genome of *D. hydrothermale*. Results are given in Supplementary Materials （Table S1). Gene clusters encoding the components of the bacterial flagellum vary greatly in their numbers and contents from one phylum to another [53]. In this genome, all flagellar proteins described to be present in all flagellated bacterial species [53] are encoded, suggesting that the flagellar apparatus is complete. The only filament protein that was not predicted was FliC, but instead, a *flaA* gene (LGS26_05105) encoding flagellin was predicted with UniProtKB. Otherwise, all central proteins of all parts of the flagellar apparatus were predicted: the hook-filament junction proteins (FlgK (LGS26_00025) and FlgL (LGS26_00020)), the hook proteins (FlgE (LGS26_00725)), the rod proteins (FlgB (LGS26_00295), FlgC (LGS26_00290), FlgG (LGS26_00055), and FlgF (LGS26_00060)), the M-ring proteins (FliF (LGS26_00280)), the flagellar motor switch protein (FliG (LGS26_00275), FliM (LGS26_08335), and FliN (LGS26_08340)). Genes encoding motor proteins were also found by protein sequence homology with *Dissulfuribacter thermophilus*, namely *motA* (LGS26_09330) and *motB* (LGS26_07810). Genes coding for flagellar biosynthesis factors and export apparatus proteins were also predicted by protein sequence homology with *Dissulfurirhabdus thermomarina*: *flhA* (LGS26_08370), *flhB* (LGS26_08365), *fliI* (LGS26_00265), *fliP* (LGS26_08350), *fliR* (LGS26_08360), *fliQ* (LGS26_08355) and *flgD* (LGS26_00720). These results are congruent with the motile character of the strain.

### 3.7. Putative Secretion Systems

Numerous genes encoding proteins of the secretion systems were predicted in the genome of *Dissulfurimicrobium hydrothermale* Sh68^T^, particularly for the type II secretion system. Few proteins of the type III secretion system were also present, but this system was incomplete. Results are detailed in Supplementary Materials (Table S2).

We studied secretion systems encoded in the genomes of 14 other S^0^-disproportionating bacteria available online at NCBI, and those of eight bacterial strains that lack the ability to disproportionate S^0^. Interestingly, a short protein (referenced as EscU/YscU/HrcU family type III secretion system export apparatus switch protein in PGAP, but whose function has not been curated and validly demonstrated) is present in the genomes of all microorganisms capable of S^0^ disproportionation (Table 2). Conversely, this short protein of unknown function is absent from all genomes of microorganisms unable to perform S^0^-disproportionation (Table 2), with the exception of the genome of *Thermodesulfatator indicus*, which encodes this short protein but whose ability to perform S^0^-disproportionation has not been experimentally demonstrated.

This short protein is composed of 89 to 122 amino acids in length in the genomes studied here. It is 94 amino acids in length in the genome of *D. hydrothermale* Sh68^T^ (locus tag LGS26_00065). It is annotated as an EscU/YscU/HrcU family type III secretion system export apparatus switch protein by PGAP but has no correspondence with the proteins referenced in the manually annotated high quality database UniProtKB providing reliable functional annotation of proteins. According to automatic annotation by PGAP, this protein could be an export apparatus switch protein involved in the type III secretion system [54], but a functional characterization will be necessary to confirm this assumption. This protein shares sequence homologies with the flagellar biosynthesis protein FlhB that is encoded in all genomes of the strains used in this comparative study but is much shorter (in *Dissulfurimicrobium hydrothermale*, the short protein and FlhB share 49.38% homology at the aligned portions). The flagellar biosynthesis protein FlhB found in these genomes is approximately 353 to 391 amino acids in length, so about three to four times longer. The secretion systems are known to share sequence homologies with flagellar proteins [54,55].

Furthermore, the presence of the short protein of unknown function does not correlate with the motility of the microorganisms studied. Indeed, some microorganisms have this protein and are not motile, and others do not have this short protein and are motile (Table 2). This protein could code for an enzyme involved in elemental sulfur disproportionation because when this protein is absent, strains have been described as unable to perform this reaction. However, this protein is also encoded in the genome of *Thermodesulfatator indicus* DSM 15286^T^ for which the ability to perform elemental sulfur disproportionation has not been described so far. Interestingly, this short protein predicted by automatic annotation software to belong to the type III secretion system is encoded in the genome of the Gram-positive S^0^-disproportionator *Dethiobacter alkaliphilus* AHT1^T^, whereas type III secretion systems are theoretically associated with Gram-negative bacteria, which might suggest that this protein plays another role in this strain [56].

### 3.8. Probable Role of the Short Protein in ISC-Disproportionation

The oxidative branch of ISC-disproportionation and the first step of S^0^ disproportionation are the least understood biochemical mechanisms of the ISC-disproportionation process. Solid elemental sulfur is poorly soluble in water (about 5 μg L^−1^ at 20 °C) [10], and the cytoplasmic membrane is impermeable to it [9].

Studies carried out with two S^0^-disproportionating bacteria, *Thermosulfurimonas dismutans* and *Desulfurella amilsii*, have demonstrated that a direct cell contact with elemental sulfur is not strictly required to perform the S^0^ disproportionation and/or S^0^ respiration but is beneficial to them [9,10]. Nevertheless, it was also observed that in *D. amilsii*, cells tend to grow close to sulfur particles and that proteomes generated under S^0^ disproportionation conditions show overexpression of proteins associated with flagella and pili systems, suggesting a possible role of these organelles in the interaction with S^0^ [10]. Different mechanisms are proposed in the literature that could allow the uptake of S^0^ by cells [10,57]. Elemental sulfur could be converted by nucleophilic attack, to a more soluble and bioavailable form of sulfur, such as polysulfides, which could then be used as an energetic substrate for S^0^ disproportionation [9,10,57]. Alternatively, elemental sulfur could form nanosulfur particles, which could directly enter the membranes [10,58]. It has also been proposed that the interaction between insoluble sulfur and thiol groups on the outer membranes could produce a soluble form of sulfur species (linear polysulfanes) [9,10]. Finally, it has also been suggested that flagella and pili may play a role in this process, either by promoting sulfur immobilization by flagella, or by allowing extracellular electron transport or direct S^0^ uptake by pili [10]. Comparative proteomic studies conducted by other authors [10] showed that some proteins belonging to pili and flagellar systems were overexpressed in the S^0^ disproportionation condition. With one exception, all genes encoding these overexpressed proteins are present in the genome of *D. hydrothermale*, including *fliD* (LGS26_05110), *flaA* (LGS26_05105), *flaG* (LGS26_05125), *flgK* (LGS26_00025), *flgL* (LGS26_00020) and *flhA* (LGS26_08370) from the flagellar apparatus, and *pilT* (LGS26_05475; LGS26_05470), *pilM* (LGS26_06170), *pilC* (LGS26_02890), and *pilB* (LGS26_04970)*,* from the pilus system (Table S1, Table S3). Only the gene coding for another overexpressed protein, namely PilQ, is not encoded in the genome of *D. hydrothermale*. These data support the hypothesis that pili and flagellar systems may play a role in S^0^ disproportionation.

The short protein of unknown function (automatically annotated as an EscU/YscU/HrcU family type III secretion system export apparatus switch protein) identified in this work, which is consistently encoded in all genomes of S^0^ disproportionators and absent in most genomes of non-S^0^-disproportionators, might be involved in the first step of S^0^ disproportionation, either by transforming S^0^ into a more soluble form, or by immobilizing it, or by extracting its electrons. We can hypothesize that this short protein may act in combination with the flagellar or pili proteins.

It is quite difficult to find specific genetic markers for sulfur disproportionation, as the ability to disproportionate inorganic sulfur compounds is not systematically investigated and reported in the characterization of sulfate reducers. As a result, the number of identified sulfur-disproportionating taxa remains limited. In addition, it is quite probable that there are several different pathways of ISC-disproportionation, which complicates the interpretation of comparative genomics data.

## 4. Conclusions

*Dissulfurimicrobium hydrothermale*, which belongs to the phylum *Desulfobacterota*, is one of the two strains identified to date that derives its energy and growth solely from the disproportionation of inorganic sulfur compounds. Although this strain is not able to grow by sulfate reduction and is unable to oxidize hydrogen, its genome encodes the enzymatic arsenal necessary to do so, i.e., a complete dissimilatory sulfate reduction pathway, as well as hydrogenases. Its autotrophic growth is possible due to the presence of all enzymes of the Wood–Ljungdhal pathway for carbon dioxide fixation. This strain, which can grow by disproportionation of elemental sulfur, sulfite and thiosulfate, encodes several proven or candidate enzymes of the ISC-disproportionation pathways: some enzymes of the sulfate reduction pathway, the YTD gene cluster, several molybdopterin oxidoreductases, a rhodanese sulfurtransferase, and numerous flagellar system proteins and pili system proteins. In addition, this study identified a short protein of unknown function that could potentially be involved in the disproportionation of elemental sulfur.

## Figures and Tables

**Table 1:** General genomic features (including MIGS mandatory information), taxonomic affiliation, and main physiological characteristics of *Dissulfurimicrobium hydrothermale* strain Sh68^T^.

Item	Description
**Investigation**	
Strain	*Dissulfurimicrobium hydrothermale Sh68^T^*
Submitted to INSDC	GenBank
Investigation type	Bacteria
Project name	PRJNA769390
Geographic location (latitude and longitude)	54°49.4′ N, 160°01.0′ E
Geographic location (country and/or sea, region)	Uzon Caldera, Kamchatka, Russia
Collection date	September 20009
Environment (biome)	Hot spring ENVO:00000051
Environment (feature)	Hot spring ENVO:00000051
Environment (material)	Hydrothermally influenced sediment ENVO:01001821
Depth	30 cm
**General features**	
Classification	Domain: *Bacteria*
	Phylum: *Desulfobacterota*
	Class: *Dissulfuribacteria*
	Order: *Dissulfuribacterales*
	Family: *Dissulfuribacteraceae*
	Genus: *Dissulfurimicrobium*
	Species: *Dissulfurimicrobium hydrothermale*
	Sh68^T^
Gram stain	Negative
Cell shape	short rod with rounded ends
Motility	Motile
Growth temperature	30–65 °C
Relationship to oxygen	Anaerobic
Trophic level	Chemolithoautotrophic
Biotic relationship	free-living
Isolation and growth conditions	DOI 10.1099/ijsem.0.000828
**Sequencing**	
Sequencing technology	Illumina Miseq Nano 2 × 150 bp and Oxford MinION (R9 flow cell and Rapid Sequencing kit)
Sequencing platform	Fasteris and in house
Assembler	Unicycler (v0.4.9)
Contig number	1
N50	2,025,450
Genome coverage	50.6 × (based only on short reads)
	131.1 × (based on short and long reads)
Genome Accession NCBI	CP085041
Assembly level	Complete
**Genomic features**	
Genome size (bp)	2,025,450
GC content (%)	49.66
Protein coding genes	1925
Number of RNAs	54
tRNAs	47
16S-23S-5S rRNAs	1-1-1

**Table 2:** Characteristics of the strains whose genomes were compared, in terms of mobility, ability to disproportionate S^0^, encoding of a small protein of unknown function, and locus tag of this protein of interest. Legend: − absence or inability; + presence or ability.

Strain	S^0^ Disproportionation Ability	Short Protein of Unknown Function (Automatically Annotated as an “EscU/YscU/HrcU Family Type III Secretion System Export Apparatus Switch Protein”)	Locus Tag of the Short Protein	Motility
*Dissulfurimicrobium hydrothermale* Sh68^T^	+	+	LGS26_00065	+
*Dissulfuribacter thermophilus* S69^T^	+	+	DBT_RS04205	+
*Thermosulfurimonas marina* SU872^T^	+	+	FVE67_RS02390	+
*Thermosulfurimonas dismutans* S95^T^	+	+	TDIS_RS03420	+
*Dissulfurirhabdus thermomarina* SH388^T^	+	+	HCU62_RS02240	+
*Caldimicrobium thiodismutans* TF1^T^	+	+	THC_RS00840	+
*Desulfobulbus propionicus* DSM 2032^T^	+	+	DESPR_RS10825	−
*Desulfofustis glycolicus* DSM 9705^T^	+	+	BUC26_RS20670	+
*Desulfocapsa sulfoexigens* DSM 10523^T^	+	+	UWK_RS15510	+
*Desulfurella amilsii* TR1^T^	+	+	DESAMIL20_RS08330	+
*Desulfurivibrio alkaliphilus* AHT 2^T^	+	+	DAAHT2_RS11940	−
*Dethiobacter alkaliphilus* AHT 1^T^	+	+	DEALDRAFT_RS03220	+
*Thermosulfuriphilus ammonigenes* ST65^T^	+	+	G4V39_RS06430	−
*Thermodesulfatator atlanticus* DSM 21156^T^	+	+	H528_RS0110240	+
*Dissulfurispira thermophila* T55J^T^	+	+	JTV28_RS00420	+
*Thermodesulfatator indicus* DSM 15286^T^	−	+	THEIN_RS08670	+
*Desulfonatronospira thiodismutans* ASO3-1^T^	−	−	−	+
*Desulfonatronum lacustre* DSM 10312^T^	−	−	−	+
*Desulfonatronum thioautotrophicum* ASO4-1^T^	−	−	−	+
*Desulfonatronum thiodismutans* MLF1^T^	−	−	−	+
*Desulfonatronum thiosulfatophilum* ASO4-2^T^	−	−	−	+
*Desulfolutivibrio sulfodismutans* DSM 3696^T^	−	−	−	+
*Desulforhopalus vacuolatus strain* DSM 9700^T^	−	−	−	−

## Data Availability

Not applicable.

## Supplementary Materials

The following supporting information can be downloaded at: https://www.mdpi.com/article/10.3390/microorganisms10010060/s1, Table S1. List of CDSs present in *D. hydrothermale* genome that are predicted to belong to the flagellar apparatus. Table S2. List of CDSs present in *D. hydrothermale* genome that are predicted to belong to the secretion systems. Table S3. List of CDSs present in *D. hydrothermale* genome that are predicted to belong to the pili systems.

## Abbreviations

AQDS	9,10-anthraquinone 2,6-disulfonate
APS	adenosine phosphosulfate
CDS	coding DNA sequences
COG	clusters of orthologous groups of proteins
DCT	DsrC-Trisulfide
ISC	inorganic sulfur compounds
KEGG	Kyoto Encyclopedia of Genes and Genomes
MIGS	minimum information about a genome sequence
NCBI	National Center for Biotechnology Information
PGAP	prokaryotic genome annotation pipeline
SDB	sulfur-disproportionating bacteria
SOR	sulfur oxygenase reductase
TCA	tricarboxylic acid cycle
