# Supplementary material for: Genetic Potential of Dissulfurimicrobium hydrothermale, an Obligate Sulfur-Disproportionating Thermophilic Microorganism
Source: Microorganisms. 2021 Dec 28;10(1):60. doi: 10.3390/microorganisms10010060 (PMC8780430; doi:10.3390/microorganisms10010060)
Supplement: Supplementary file 1 [file microorganisms-10-00060-s001.zip › Supplementary_Materials_Dhydrothermale-OK.pdf]

Complete genome sequence analysis of *Dissulfurimicrobium hydrothermale*, a thermophilic sulfur-disproportionating microorganism

Stéven Yvenou[1], Maxime Allioux[1], Alexander Slobodkin[2], Galina Slobodkina[2], Mohamed Jebbar[1] and Karine Alain[1]*

[1]Univ Brest, CNRS, IFREMER, IRP 1211 MicrobSea, Laboratoire de Microbiologie des Environnements Extrêmes LM2E, IUEM, Rue Dumont d'Urville, F-29280 Plouzané, France

[2]Winogradsky Institute of Microbiology, Research Center of Biotechnology of the Russian Academy of Sciences, Moscow, Russia

*Correspondence: Karine.Alain@univ-brest.fr

Supplementary materials:

Table S1. List of CDSs present in *Dissulfurimicrobium hydrothermale* Sh68^T genome that are predicted to belong to the flagellar apparatus.

Protein predicted by MaGe, PGAP, and/or PROKKA	Locus tag
flagellar assembly protein FliW	LGS26_00010
flagellar hook-associated protein FlgL	LGS26_00020
flagellar hook-associated protein FlgK	LGS26_00025
flagellar basal body P-ring protein FlgI	LGS26_00040
flagellar basal body L-ring protein FlgH	LGS26_00045
flagellar basal body P-ring formation chaperone FlgA	LGS26_00050
flagellar basal-body rod protein FlgG	LGS26_00055
flagellar basal-body rod protein FlgF	LGS26_00060
flagellar hook-length control protein FliK	LGS26_00070
flagellar export protein FliJ	LGS26_00260
flagellar motor switch protein FliG	LGS26_00275
flagellar M-ring protein FliF	LGS26_00280
flagellar hook-basal body complex protein FliE	LGS26_00285
flagellar basal body rod protein FlgC	LGS26_00290
flagellar basal body rod protein FlgB	LGS26_00295
flagellar hook-length control protein FliK	LGS26_00715
flagellar hook protein FlgE	LGS26_00725
flagellar filament capping protein FliD	LGS26_05110
flagellar protein FliS	LGS26_05115
flagellar protein FlaG	LGS26_05125
flagellar biosynthesis anti-sigma factor FlgM	LGS26_07230
flagellar basal body-associated FliL family protein	LGS26_08330
flagellar motor switch protein FliM	LGS26_08335
flagellar motor switch protein FliN	LGS26_08340
flagellar biosynthetic protein FliO	LGS26_08345
flagellar type III secretion system pore protein FliP	LGS26_08350
flagellar biosynthesis protein FliQ	LGS26_08355
flagellar biosynthetic protein FliR	LGS26_08360
flagellar biosynthesis protein FlhB	LGS26_08365
flagellar biosynthesis protein FlhA	LGS26_08370
flagellar motor stator protein MotA	LGS26_09330
FliI/YscN family ATPase	LGS26_00265
Flagellar basal-body rod modification protein FlgD	(LGS26_00720)

Table S2. List of CDSs present in *Dissulfurimicrobium hydrothermale* Sh68^T genome that are predicted to belong to the secretions systems.

**Protein predicted by MaGe, PGAP, and/or PROKKA**	**Locus tag**
EscU/YscU/HrcU family type III secretion system export apparatus switch protein	LGS26_00065
type II secretion system GspH family protein	LGS26_01270
type II secretion system major pseudopilin GspG	LGS26_01680
type II secretion system ATPase GspE	LGS26_01685
type II secretion system F family protein	LGS26_01690
type II secretion system GspH family protein	LGS26_01700
general secretion pathway protein GspK	LGS26_01710
type II secretion system secretin GspD	LGS26_01725
HlyD family secretion protein	LGS26_04385
type II secretion system F family protein	LGS26_04975
flagellar type III secretion system pore protein FliP	LGS26_08350
type II secretion protein	LGS26_08815

Table S3. List of CDSs present in *Dissulfurimicrobium hydrothermale* Sh68^{T} genome that are predicted to belong to the pili systems.

**Protein predicted by MaGe, PGAP, and/or PROKKA**	**Locus tag**
PilT/PilU family type 4a pilus ATPase	LGS26_05470
type IV pilus twitching motility protein PilT	LGS26_05475
PilZ domain-containing protein	LGS26_05835
pilus assembly protein PilM	LGS26_06170
PilN domain-containing protein	LGS26_06175
type 4a pilus biogenesis protein PilO	LGS26_06180
pilus assembly protein PilP	LGS26_06185
pilus (MSHA type) biogenesis protein MshL	LGS26_06195
PilZ domain-containing protein	LGS26_07805
prepilin-type N-terminal cleavage/methylation domain-containing protein	LGS26_01270
type II secretion system major pseudopilin GspG	LGS26_01680
prepilin-type N-terminal cleavage/methylation domain-containing protein	LGS26_01695
prepilin-type N-terminal cleavage/methylation domain-containing protein	LGS26_01705
PilN domain-containing protein	LGS26_01715
PilZ domain-containing protein	LGS26_02065
prepilin-type N-terminal cleavage/methylation domain-containing protein	LGS26_02310
prepilin-type N-terminal cleavage/methylation domain-containing protein	LGS26_02315
Flp pilus assembly complex ATPase component TadA	LGS26_03090
pilus assembly protein PilX	LGS26_03335
prepilin-type N-terminal cleavage/methylation domain-containing protein	LGS26_03340
prepilin-type N-terminal cleavage/methylation domain-containing protein	LGS26_03345
prepilin-type N-terminal cleavage/methylation domain-containing protein	LGS26_03350
pilus (MSHA type) biogenesis protein MshL	LGS26_05000
Type IV pilus assembly protein PilC	LGS26_02890
Type IV fimbrial assembly, ATPase PilB	LGS26_04970
